# Balance Trees Reveal Microbial Niche Differentiation

**DOI:** 10.1128/mSystems.00162-16

**Published:** 2017-01-17

**Authors:** James T. Morton, Jon Sanders, Robert A. Quinn, Daniel McDonald, Antonio Gonzalez, Yoshiki Vázquez-Baeza, Jose A. Navas-Molina, Se Jin Song, Jessica L. Metcalf, Embriette R. Hyde, Manuel Lladser, Pieter C. Dorrestein, Rob Knight

**Affiliations:** a Department of Pediatrics, University of California San Diego, La Jolla, California, USA; b Department of Computer Science and Engineering, University of California San Diego, La Jolla, California, USA; c Collaborative Mass Spectrometry Innovation Center, Skaggs School of Pharmacy, University of California San Diego, La Jolla, California, USA, and Department of Animal Sciences, Colorado State University, Fort Collins, Colorado, USA; d Department of Applied Mathematics, University of Colorado Boulder, Boulder, Colorado, USA; e Pharmaceutical Sciences, University of California San Diego, La Jolla, California, USA; Pacific Northwest National Laboratory

**Keywords:** Aitchison geometry, balance trees, compositionality, cystic fibrosis, niche, soil microbiology

## Abstract

Advances in sequencing technologies have enabled novel insights into microbial niche differentiation, from analyzing environmental samples to understanding human diseases and informing dietary studies. However, identifying the microbial taxa that differentiate these samples can be challenging. These issues stem from the compositional nature of 16S rRNA gene data (or, more generally, taxon or functional gene data); the changes in the relative abundance of one taxon influence the apparent abundances of the others. Here we acknowledge that inferring properties of individual bacteria is a difficult problem and instead introduce the concept of balances to infer meaningful properties of subcommunities, rather than properties of individual species. We show that balances can yield insights about niche differentiation across multiple microbial environments, including soil environments and lung sputum. These techniques have the potential to reshape how we carry out future ecological analyses aimed at revealing differences in relative taxonomic abundances across different samples.

**IMPORTANCE** By explicitly accounting for the compositional nature of 16S rRNA gene data through the concept of balances, balance trees yield novel biological insights into niche differentiation. The software to perform this analysis is available under an open-source license and can be obtained at https://github.com/biocore/gneiss.

**Author Video**: An author video summary of this article is available.

## Abstract (precis)

By explicitly accounting for the compositional nature of 16S rRNA gene data through the concept of balances, balance trees yield novel biological insights into niche differentiation. The software to perform this analysis is available under an open-source license and can be obtained at https://github.com/biocore/gneiss.

## INTRODUCTION

The ultimate goal for many microbial ecologists is to fully characterize niches of microbial organisms and understand interactions among taxa. An understanding of how microbial communities are affected by environmental conditions might yield insights into microbial interactions and their role in macroecological processes, such as nitrogen fixation (1) and acidification (2). However, despite the extraordinary increase in available data brought about by advances in DNA sequencing, characterizing niche differentiation in microbes remains an outstanding problem, partly due to the difficulty of correctly interpreting compositional data. Broadly speaking, a compositional data set is represented by relative abundances, or proportions, that individually carry no meaning for the absolute abundance of a specific feature (i.e., 20% of 100 and 20% of 10,000 are very different absolute abundances). The constraints associated with compositional data are well known but unfortunately often neglected in microbial ecology, leading to conflicting interpretations and irreproducible analyses (3, 4).

**FIG 1:**
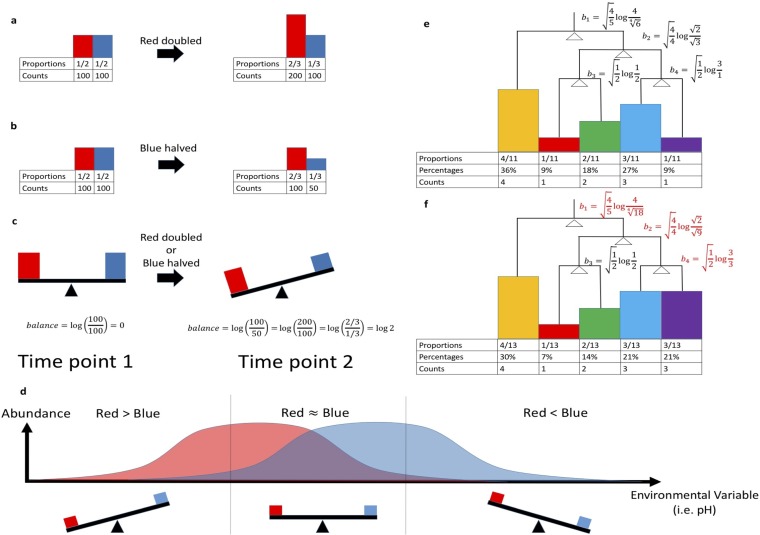
(a, b) Hypothetical scenario where 2 samples of 2 proportions may explain two different scenarios in the environment. (c) The balance between these 2 proportions is consistent for both scenarios. (d) Balance of Red and Blue species abundances. (e, f) Balances of Red and Blue individuals across an environmental variable. The comparison is of proportions and balances of two environments in the scenario where the Purple/Orange population (i.e., the most-right bin) triples. The balances were calculated using the groupings specified by the tree.

We illustrate an example of this problem in Fig. 1. In this scenario, there are two species, “Red” and “Blue.” At the first time point, there are 100 Red individuals and 100 Blue individuals (Fig. 1a). At the next time point, the number of Red individuals doubles, yielding 200 Red individuals, and the proportions of Red and Blue individuals become two-thirds and one-third, respectively (Fig. 1b). Suppose that we do not know the true total number of individuals in the given environment and can only make inferences about the observed proportions, a common scenario in microbial ecology, where absolute quantification is rarely performed. In Fig. 1b, the community has the exact same proportions at time 1 and time 2 as those in Fig. 1a; however, instead of the Red individuals doubling at the second time point, the number of Blue individuals is halved (Fig. 1c).

This is the problem with compositionality; based on proportions alone, it is impossible to determine whether the growth or decline of any individual species has truly occurred (5), and the inherent feature of one change in abundance driving abundance changes in another species violates assumptions of independence. Analyses that rely on such assumptions, as many statistical approaches do, are thus prone to misinterpretation. For example, traditional correlation metrics, such as Pearson and Spearman metrics, can be misleading when estimating microbe-microbe correlations (6–9). As a result, it becomes a major challenge to specify types of interactions between microbes, such as parasitism, competition, predation, or mutualism, as shown in correlation studies of oral, fecal, and vaginal samples from the Human Microbiome Project (6, 10). Even-more-advanced correlation-detection techniques, such as SparCC (3) and SPIEC-EASI (5), struggle with this and typically require additional assumptions, such as sparse operational taxonomic unit (OTU) correlations (i.e., few OTUs are actually correlated with each other). Furthermore, interpreting the resulting network is a major challenge, making it difficult to differentiate between true ecological relationships and random processes (10).

The compositionality problem is also problematic for statistically detecting differentially abundant microbes across environments or between groups; consequently, it is a major barrier to reliably drawing conclusions about realized microbial niches using community sequencing data. Conventional statistical tools, such as the *t* test and Mann-Whitney test, can incorrectly identify nearly 100% of the taxa present in samples to be significantly different across environments (see Fig. S1 in the supplemental material), and univariate tests, such as *t* tests and zero-inflated Gaussian (ZIG)-based methods (11), have been shown to mislabel microbes as significantly different across sample groups up to 60% of the time (12). More-advanced tools for differential abundance detection, such as analysis of compositions of microbiomes (ANCOM) (12), are typically designed to control for false-positives and reliably detect differentially abundant species, but they require multiple assumptions (i.e., the number of changing microbes across environments is small) and may require complex parameter tuning. To help overcome these issues of compositionality, we explore using the concept of balances by moving away from inferring changes of individual species to instead inferring changes of microbial subcommunities to study the niche differentiation of microbial communities.

10.1128/mSystems.00162-16.1 FIG S1  (a) False-discovery rate (FDR) of a *t* test used on proportions in the presence of 1 blooming species in a uniform population; (b) false-discovery rate of a Mann-Whitney *U* test on proportion; (c) false-discovery rate of a *t* test on balances with the tree shown in panel e; (d) false-discovery rate of a *t* test on balances with the tree shown in panel g; (e) distribution of *t* test *P* values on proportions; (f) tree with 1,000 tips used for the balance test, with the blooming species being ×1; (g) different tree used for the balance test, where the blooming species was placed on the bottom of the tree. Download FIG S1, PDF file, 0.2 MB. Copyright © 2017 Morton et al. 2017 Morton et al. This content is distributed under the terms of the Creative Commons Attribution 4.0 International license.

## CONCEPT

Balances were first introduced as an exploratory technique in geology (13, 14). Fundamentally, they overcome the problem of inferring changes in abundance from compositional data by sidestepping it and by instead inferring changes in the balance between particular subsets of the community. To understand the concept, let us revisit the scenario in Fig. 1a and b. Instead of examining proportion changes, we can investigate the balance between Red and Blue individuals by taking the log ratio of Red and Blue counts (Fig. 1c). By looking at the balance of these two species, we avoid incorrectly attempting to infer absolute increases or decreases in their abundances. Instead, we can focus on the balance of the Red and Blue individuals and directly infer the transition of dominance between these species.

These balances can also be useful for understanding species distributions across different covariates, a key proximate goal of microbial ecology and one that is both crucial to the larger goal of niche characterization and heavily impacted by problems inherent in compositionality. In Fig. 1d, the Red individuals tend to exist at the low-pH end of the spectrum, while the Blue individuals tend to exist at the high-pH end of the spectrum. A single balance can capture information about the transition from a high relative abundance of Red individuals in low-pH environments to a high relative abundance of Blue individuals in high-pH environments. In low-pH environments, the balance is positive, since there are proportionally more Red individuals than Blue individuals. When the Red and Blue individuals are present in roughly equal proportions, the balance is roughly zero, representing a turning point, transitioning from a Red species-dominated community to a Blue species-dominated community. As the pH increases, the balances become increasingly negative, since there are more Blue individuals than Red individuals. This balance effectively encodes the niche separation of Red and Blue individuals across the pH gradient.

This idea of balances can be extended to multiple dimensions—and to more than two taxa—using bifurcating trees. A bifurcating tree can be built relating microbial taxa to each other by using any criterion, and balances can be calculated on the internal nodes of the tree from the geometric means of the corresponding subtrees. The appropriate criterion to build a tree depends on the question at hand. A phylogenetic tree could be used to investigate evolutionary relationships of microbes (15, 16), or hierarchical clustering of environmental variables could be used to explore environmental niches of microbes. To gain more intuition about this, consider Fig. 1e, in which there are five species and 11 individuals. The four balances (internal nodes in the tree) are calculated by taking the log ratio of geometric means of subtrees, also known as the isometric log ratio (ILR) transform. The full equation to calculate balances for a single sample is as follows:

(1) $$b_{i}=\sqrt{\frac{\left|i_{L}\right|\left|i_{R}\right|}{\left|i_{L}\right|+\left|i_{R}\right|}}\log\left[\frac{g(i_{L})}{g(i_{R})}\right]$$

where *b_i_* is the balance at internal node *i*, *i_L_* is the set of all species proportions contained in the left subtree at internal node *i*, *i_R_* is the set of all species proportions contained in the right subtree at the internal node *i*, *g*(*x*) is the geometric mean of all of the proportions contained in vector *x*, |*i_R_*| is the number of species contained in *i_R_*, and |*i_L_*| is the number of species contained in *i_L_* (see Materials and Methods for more details). According to this equation, in Fig. 1f, *b*_1_ is calculated by taking the log ratio of the Yellow species and the geometric mean of the Red, Green, Blue, and Purple species.

It is also important to note that some of the balances do not impact each other. For instance, the changes in *b*_4_ do not impact the changes in* b*_3_, just because these balances do not share any common tips. This is crucial, because this property allows us to ignore some of the variance of the balances toward the tips of the tree and to focus on the balances closer to the root of the tree. These balances toward the root of tree capture the most information, since they contain a significant proportion of tree tips. As a result, these high-level balances have the potential to explain large shifts in these microbial communities. The choice of the tree can allow for analysts to embed prior knowledge into the structure of the tree to test for these large community shifts.

Here, we will discuss two studies from which novel insights were gained from this application. While many compositionally aware tools that are designed to identify microbial interactions and abundance fluctuations are available, we will refrain from benchmarking balances against these tools, as balances answer a conceptually different question. These analyses are not restricted to analyzing ratios of individual OTUs and can easily be extended to analyze ratios of subcommunities.

## RESULTS

### Case study 1: balances of pH-driven subcommunities in soils.

In this study (17), 88 soil samples were collected from North and South America, along with many edaphic measurements. The study reported that there was a strong correlation between pH and species richness, suggesting that pH was a strong driver behind fluctuations in soil microbial communities. *Acidobacteria* were found to be negatively correlated with pH and *Actinobacteria* and *Bacteroidetes* to be positively correlated with pH, while alpha-, beta-, and gammaproteobacteria were not correlated with pH at all. These correlation analyses are a little misleading, since the pH was correlated with each of the phyla independently. The problem with this approach is that it does not account for all of the other phyla; as with the argument made for the experiment represented in Fig. 1b, the change in a single phylum might also be explained by correlated changes in all of the other phyla. Here, the negative correlation between *Acidobacteria* abundance and pH might also be caused by the positive correlation between *Bacteroidetes* abundance and pH. Additionally, we cannot determine whether the alpha-, beta-, and gammaproteobacteria are correlated with pH or not. Another possibility is that these three phyla are positively correlated with pH but that *Acidobacteria* abundance is not correlated with pH. However, *Bacteroidetes* may be so strongly correlated with pH that *Acidobacteria* appear to be negatively correlated with pH and the other three phyla to not be correlated with pH at all. This scenario is one of the infinite possible underlying relationships that can explain these observed correlations.

**FIG 2:**
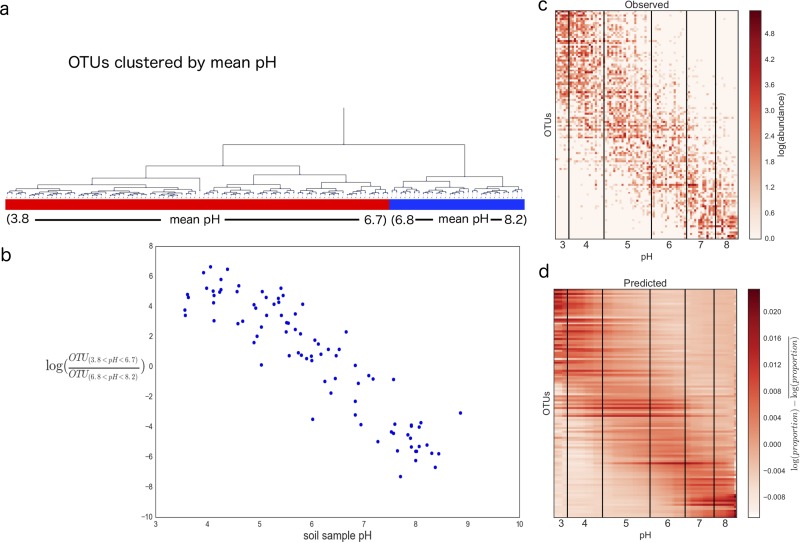
(a) Hierarchical clustering of closed-reference OTUs based on mean pH; (b) balance of low-pH-associated organisms (3.8 < mean pH < 6.7) and high-pH-associated organisms (6.8 < mean pH < 8.2); (c) observed OTU counts sorted by pH; (d) predicted OTU proportions from ordinary least-squares linear regression on balances sorted by pH. The coefficient of determination was 35%, showing that 35% of the variation in the microbial community abundance data can be predicted by pH alone.

At a first glance, uncovering the true correlations correctly appears to be a hopeless cause. This is where balances become useful. Rather than attempting to correlate individual phyla against pH, we grouped OTUs together according to their difference in mean pHs (Fig. 2a) and investigated how these balances of groups change with respect to pH (see Materials and Methods on hierarchical clustering). This circumvented the dependence issue noted previously. We do not need to worry about subgroups within the left and right subtrees of a balance influencing each other, due to the independence property shown in Fig. 1e and f.

The balance concept proves to be a very powerful technique for investigating how these groups of organisms change relative to each other as pH increases. Recall the cartoon example in Fig. 1d. If there are two distinct unimodal species distributions, the balance pivots from being weighted by Red individuals in low pH to being weighted by Blue individuals in high pH. The same phenomenon occurs here, except that there are multiple species on the left end of the balance and multiple species on the right end of the balance.

As shown in Fig. 2b, there is a well-defined trend of low-pH OTUs (3.8 < mean pH < 6.6) gradually being overtaken by high-pH OTUs (6.7 < mean pH < 8.2) as the pH increases, forming a nice linear trend defined by the top balance in the tree shown in Fig. 2a. If we were to sort the samples by their mean pHs and the OTUs by their mean pHs (see equation 3 in Materials and Methods), a well-defined band pattern appears. Here, it is clear that OTUs with a mean pH less than 3 rarely have nonzero counts above 8. Likewise, OTUs that have a mean pH of more than 8 rarely have nonzero counts below 3. If we tie in this band pattern in Fig. 2c together with the balance-versus-pH trends shown in Fig. 2b, we obtain a very different interpretation from that of the original study. OTUs tend to be observed in very specific pH ranges but not commonly observed outside these ranges. This ties together with some concepts in niche theory: OTUs are more suited to live within a designated range of pHs, and if they are placed outside this pH range, they are outcompeted by other organisms who are more suited to live within the given pH range.

These patterns were completely missed when we looked only at the phylum level in the original study. In fact, based on the calculated mean pH values for each OTU, it was observed that OTUs from all of the phyla mentioned in the study were widely distributed across the pH gradient (see Table S1 in the supplemental material). As an extreme example, OTUs from the family *Bradyrhizobiaceae* were observed to be present at both ends of the spectrum; some were present at pH values as low as 5.36, while others were present at a pH value as high as 6.75. These are astronomical differences, considering that 95% of the OTUs have a mean pH that falls between this range. This provides additional justification for building a tree based on mean pH rather than bacterial phylogeny.

10.1128/mSystems.00162-16.2 TABLE S1 Table of OTUs, their taxonomies, and the mean of the environmental pH values in which they were observed. Download TABLE S1, XLSX file, 0.1 MB. Copyright © 2017 Morton et al. 2017 Morton et al. This content is distributed under the terms of the Creative Commons Attribution 4.0 International license.

Finally, these balances can be used to build predictive models. Using ordinary least-squares analyses on the calculated balances, the entire microbial community profile can be predicted using pH alone with an *R*^2^ of 0.35. This means that pH alone explains over 35% of the total variation in entire soil microbial communities across North and South America. The resulting fit can be transformed back to proportions to yield the predicted proportions (Fig. 2d). From this heatmap, the key patterns, such as the band pattern apparent in Fig. 2c, are still retained. There are many published regression techniques that attempt to use microbial abundances to predict covariates, such as the postmortem interval (18) or body mass index (19). This approach is the first of its kind to attempt to address the reverse problem: to predict entire microbial community distribution based on environmental variables. These predictions were enabled by the powerful fundamental properties of balances.

### Case study 2: balances of pH-driven subcommunities in a lung sputum culture microcosm.

In this study, lung sputum samples were collected from 16 cystic fibrosis (CF) patients. These sputum samples were then grown in a capillary tube culture system (Winogradsky cystic fibrosis system) that mimics the conditions of a lung bronchiole (20). These samples were placed into separate tubes, and the pH of the media was adjusted from 5 to 8.5 at intervals of 0.5 to determine how the microbial community changed with respect to pH. After growth in the capillary tubes, the communities were assessed using 16S rRNA gene amplicon sequencing.

**FIG 3:**
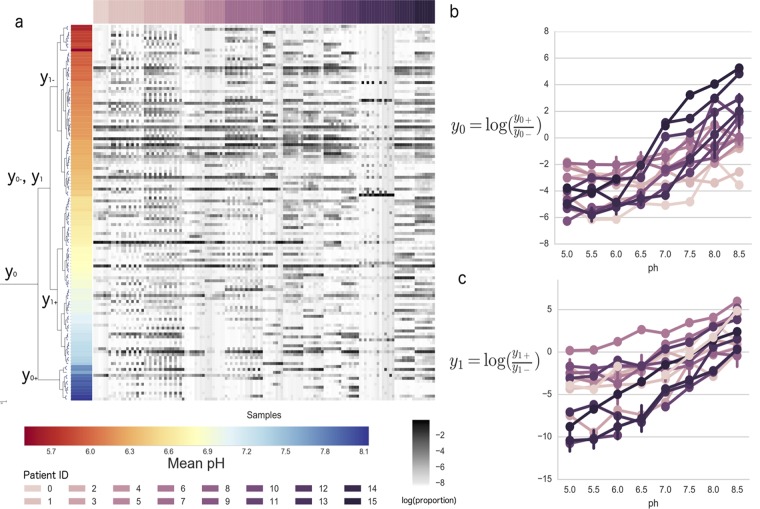
(a) Bifurcating tree generated from hierarchical clustering of OTUs based on mean pH. The size of the internal nodes is inversely proportional to the *P* value of the linear mixed-effects model test on pH for that given balance. A heatmap of all of the OTU abundances sorted by patient is shown. OTUs were log transformed and centered across rows and columns. These abundances are aligned with the tips of the tree. (b) Progression of the top balance over the pH for all of the patients. (c) Progression of the second top balance over the pH for all of the patients.

One of the difficulties in this study was characterizing pathogenic bacteria. Early on in this case study, the only significant finding discovered was that patients had different lung sputum microbiomes (Fig. 3a). It was hypothesized that there was a subcommunity of low-pH organisms and a subcommunity of high-pH organisms that periodically appeared and disappeared in CF lung sputum. However, these changes could not be detected using available statistics, likely due to the compositionality problem. Since the different CF patients had idiosyncratic lung communities, they ended up having different OTUs responding across the laboratory pH gradient, yielding insufficient statistical power to detect changes in any given OTU. As a result, when these lung sputum communities were placed into different media and studied, it was not clear exactly what organisms were a part of this low-pH or high-pH subcommunity.

Balances are a natural solution to this problem. In addition to probing for patterns similar to those observed in the previous study, balances are well adapted as a transformation for standard statistical analyses. Since Euclidean operations directly translate into perturbation and powering operations on proportions (21, 22), many of the publicly available statistical tools can be applied directly to balances. For this study, we opted to use linear mixed-effects models to test for pH differences while simultaneously accounting for all of the differences between lung microbiomes across CF patients. Based on prior analyses with pH in soils, the tree was built using the exact same strategy (see Materials and Methods). Significant balances from testing for pH were determined with a *P* value cutoff at 0.05 after Bonferroni correction.

A heatmap relating pH to OTU abundances across these samples does not yield clear trends (Fig. 3a), but even though we do not see a clear pattern in the heatmap with the balance approach, we can still observe niche differentiation across the pH gradient. In Fig. 3b, y0 represents the log ratio of all of the high-pH OTUs (7.6 < mean pH < 8.12) over all of the low-pH OTUs (5.4 < mean pH < 7.4). As the pH of the samples increases, the balance increases, likely because the low-pH OTUs are becoming increasingly less abundant than the high-pH OTUs (*P* value = 7.5 × 10^−46^). The same pattern is even more apparent in *y*_1_ (Fig. 3c). The low-pH OTUs (5.4 < mean pH < 6.4) become increasingly less abundant than high-pH OTUs (6.5 < mean pH < 7.4) as the sample pH increases (*P* value = 2.25 × 10^−67^). When Bonferroni multiple-hypothesis correction was applied to these tests, the *P* values were rounded down to zero. While these patterns were not obvious when looking at the raw proportions, the balance tree approach shows very well defined trends among groups of OTUs. This can be done because even though individual OTUs may be sporadically distributed across the original samples, OTUs that thrive in similar pH niches grouped together on the environmental balance tree. It is clear from Fig. 3b and c that there is a transition from low-pH organisms to high-pH organisms along the pH gradient. Even though the CF patients do not have the same lung microbiomes, they contain OTUs that behave the same with respect to pH. This pattern would not have been nearly as apparent without clustering the OTUs by mean pH and accounting for the patient effects in the linear mixed-effects models.

## DISCUSSION

In this study, we have demonstrated the benefits of applying balances to infer niche differentiation in microbes. In the first case study, we outlined the challenge of performing correlations of OTUs versus environmental variables and showed how balances can capture information about species turnover across the pH gradient, which allowed us to build a model to predict microbial proportions based on pH alone. In the second case study, we identified the challenges of studying individual OTUs due to similar niches being occupied by drastically different OTUs across different patients. Balances coupled with linear mixed-effects models allowed us to obtain more statistically robust results, which were also more informative with respect to the differences in distribution of microbes across environmental niches.

There are numerous additional benefits of analyzing species balances instead of individual species counts. First, balances are known to be scale invariant, so balance trees naturally correct for differences in sequencing depth without requiring rarefaction (see Text S1 in the supplemental material) and avoid many of the limitations associated with this procedure (23). Second, balances are subcompositionally coherent, which means that changes in nonoverlapping subcommunities do not impact each other. For instance, in Fig. 1e and f, the Purple population triples, balances, and changes because the organisms explicitly contain the Purple species. In contrast to proportions, the balance b3 does not change between these two scenarios because it does not relate to the Purple species (in fact, it accounts only for the Red and Green species). This is not the case when observing the raw proportions, from which it appears as though everything is changing, even though the Purple species is the only changing species. This phenomenon has previously been noted (12) and can lead to extremely high false-positivity rates with some standard statistical techniques, such as Pearson correlations or *t* tests on proportions. More discussion about this issue can be found in Fig. S1. Third, arithmetic operations on balances directly translate into perturbation and powering operations on proportions (21, 22), which can capture information about relative growth and decay of species. This ultimately opens the door for applying standard statistical techniques, such as multiple linear regression (24) and linear mixed-effects model nested-design statistics, directly to balances, providing additional justification for the analyses performed in the case studies. We have shown this in the two case studies. Finally, balances are permutation invariant. Species can be sorted in any order deemed appropriate. Along the same lines, these species can be rearranged into any arbitrary grouping represented as a bifurcating tree. These trees can be built to address the questions at hand, whether it be studying species turnover across pH gradients or even uncovering the relationships between phylogenetic clades. In fact, balances can be thought of as being utilized as an ordination technique, since every bifurcating tree forms an orthonormal basis in the Aitchison simplex (13).

10.1128/mSystems.00162-16.3 TEXT S1  Equation demonstrating the scale invariance property of balances. Download TEXT S1, DOCX file, 0.1 MB. Copyright © 2017 Morton et al. 2017 Morton et al. This content is distributed under the terms of the Creative Commons Attribution 4.0 International license.

Although the concept of balances does not address questions about properties of individual bacteria, it does answer higher-level questions concerning interactions among groups of organisms, which are arguably much more interesting from an ecological point of view. These questions can be based either on the phylogenetic tree of the bacterial community or on environmental clustering. There is still room for improvement on utilizing balances. For example, the issue of zeroes still remains, because the logarithm of zero is undefined. Currently, the common approach is to add a pseudocount (25). However, an appropriate tree choice can mitigate this issue, because the zeroes can be explicitly aggregated in some scenarios (Fig. S2 and S3). Along the same lines, issues can arise from low-coverage samples. If sampling is not saturated, many OTUs have low read counts, and the balances toward the tips of the trees can be highly volatile. This is because the absolute change between one or two reads may be small for low-abundance OTUs, but this will lead to large changes in log ratios, which lead to spurious signals at the tips of the tree. As a rule of thumb, balances toward the root of the tree are more trustworthy than those at the tips of the tree.

10.1128/mSystems.00162-16.4 FIG S2  (a) Density plots of 4 unimodal species distributions, with all proportions below 0.0001 rounded down to zero; (b) balances from the unimodal species distributions; (c) tree used to guide the balance calculations. Download FIG S2, PDF file, 0.1 MB. Copyright © 2017 Morton et al. 2017 Morton et al. This content is distributed under the terms of the Creative Commons Attribution 4.0 International license.

10.1128/mSystems.00162-16.5 FIG S3  (a to d) Different high-level balances versus pH in the 88 soils study; (e) same tree as that shown in Fig. 2b with some of the internal nodes labeled. Download FIG S3, PDF file, 0.1 MB. Copyright © 2017 Morton et al. 2017 Morton et al. This content is distributed under the terms of the Creative Commons Attribution 4.0 International license.

The balance approach will be key for analyzing functional roles of OTUs. It is known that in environments like the human gut, people share very few OTUs with each other but have roughly the same proportions of functional genes (26). This suggests that there is substantial functional redundancy across OTUs, which has been observed previously in time series studies in the context of infection (27); in other words, in these microbial communities, many players might be sporadically distributed across similar niches. This phenomenon might explain the sparse nature of 16S relative abundance data and why similar environments, such as human guts, share few common OTUs. Such distributions pose tremendous challenges to analyses based around identifying the niche occupancy of individual OTUs. By instead permitting the statistical comparisons to be performed across nested groups of OTUs with similar distributions, it becomes possible to robustly identify patterns of niche differentiation without requiring sufficient information to be present in the abundances of each individual taxon. Identifying common functional roles of potentially diverse organisms and analyzing the balances between these groups might significantly simplify analyses in future amplicon studies. The ability to construct such trees would enable rapid characterizations of environmental niches and the corresponding functional roles of the microbes occupying in these niches.

All in all, balance trees are an extremely powerful tool for analyzing relative abundances and uncovering patterns associated with niche differentiation, while avoiding the issues associated with compositionality and enabling the application of conventional statistical tools. This will ultimately open the door for extensive mining of ecologically relevant patterns.

## MATERIALS AND METHODS

The core functions required to perform the balance basis calculations, the tree visualization tools, and statistical analyses can be found in https://github.com/biocore/gneiss. The IPython notebooks used to carry out all of the analyses can be found in the gneiss repository. All code has been extensively unit tested and documented.

The core compositional statistics and tree data structures were are part of scikit-bio 0.4.1 and beyond. The hierarchical clustering was performed using SciPy. Pandas and BIOM (23) were used to store and manipulate the OTU tables and the metadata files. Seaborn, matplotlib, and ETE (24) were used for the visualizations.

The isometric log ratio transform is an isomorphism (i.e., a function) that can map proportions to balances one-to-one (21). These balances can be calculated as shown in equation 1. Alternatively, they can be calculated using a linear transformation with an orthonormal basis *e*.

This orthonormal basis can be calculated as follows:

(2) $$\begin{array}{l} e_{l}=C\left[\exp\underset{k}{\underset{︸}{(0,...0,}}\underset{r}{\underset{︸}{a,...,a,}}\underset{s}{\underset{︸}{b,...,b,}}\underset{t}{\underset{︸}{0,...0)}}\right]\\ a=\frac{\sqrt{s}}{\sqrt{r(r+s)}}\;\text{and}\;b=\frac{-\sqrt{r}}{\sqrt{s(r+s)}} \end{array}$$

where *e*_*l*_ refers to the balance axis aligned with the internal node l, *C*(*x*) denotes the normalization operation to normalize all of the OTU abundances to proportions that add up to 1, *r* refers to the number of tips in the left subtree, *s* refers to the number of tips in the right subtree, *k* refers to number of tips to the left of the left subtree, and *t* refers to the number of tips to the right of the right subtree. Since *e* forms an orthonormal basis, it must have unit norm, and every pair of axes in *e* must be orthogonal. The square root term in equation 1 is a normalization factor which was required for unit norm in equation 2 (12). Since it is not possible to take a logarithm of zero, a pseudocount of 1 was added to all of the abundances. While this is a problem being addressed by the field, this technique is one of the more commonly used techniques (Martín-Fernández et al. [25]).

The mean pH used for the 2 case studies was calculated as follows:

(3) $${\overset{¯}{g}}_{x}=\sum_{i\;=\;1}^{N}g_{i}\;\frac{x_{i}}{\sum_{j\;=\;1}^{D}x_{j}}$$

where *x_i_* is the proportion of OTU *x* in sample *i*, $g_{x}$ is the mean pH of OTU *x*, and *g_i_* is the sample pH at sample *i*. This calculation can be found in the gneiss package under the function mean_niche_estimator. The function used to sort the tables in Fig. 2c used niche_sort. The resulting tree was built using the unweighted pair group method using average linkages (UPGMA) (16). Results are shown in Fig. 2a and 3a and can be generated using the SciPy linkage function.

The linear regression on balances and linear mixed-effects models on balances were implemented in gneiss under the ordinary least-squares (OLS) and mixed functions, and the case study analyses can also be found in the IPython notebooks in the ipynb folder in 88soils.ipynb (case study 1) and cfstudy.ipynb (case study 2), respectively. In case study 1, only OTUs that had more than 100 reads in the entire study were considered. In case study 2, only OTUs that had more than 500 reads were considered.

The WinCF system was used according to the methods in reference 17, except only the pH dye medium variable was used. The medium was buffered at 0.5 unit of pH from 5 to 8.5 using calculated proportions of phosphate buffer and NaOH or HCl. Sputum samples were collected from CF patients after expectoration or induced expectoration of sputum according to UCSD IRB-approved project 081500 and were inoculated in triplicate into capillary tubes containing the eight different-pH buffered media. These eight sets of tubes in triplicate from 18 patients were then incubated at 37°C for 48 h. The medium was then removed, bacterial DNA was extracted, and variable region 4 of the 16S rRNA gene was amplified and sequenced on the Illumina MiSeq platform using Earth Microbiome Project-benchmarked protocols (25, 26). Data were processed using Qiita, and OTUs were calculated using closed reference clustering at the 97% identity cutoff for both the 88 soils and the CF study.

### Data availability.

Data for case study 1 were retrieved from Qiita (study identifier 103 [https://qiita.ucsd.edu/study/description/103]), as were data for case study 2 (study identifier 10511).
