# Supplementary figures and images for: Balance Trees Reveal Microbial Niche Differentiation
Source: mSystems. 2017 Jan 17;2(1):e00162-16. doi: 10.1128/mSystems.00162-16 (PMC5264246; doi:10.1128/mSystems.00162-16)

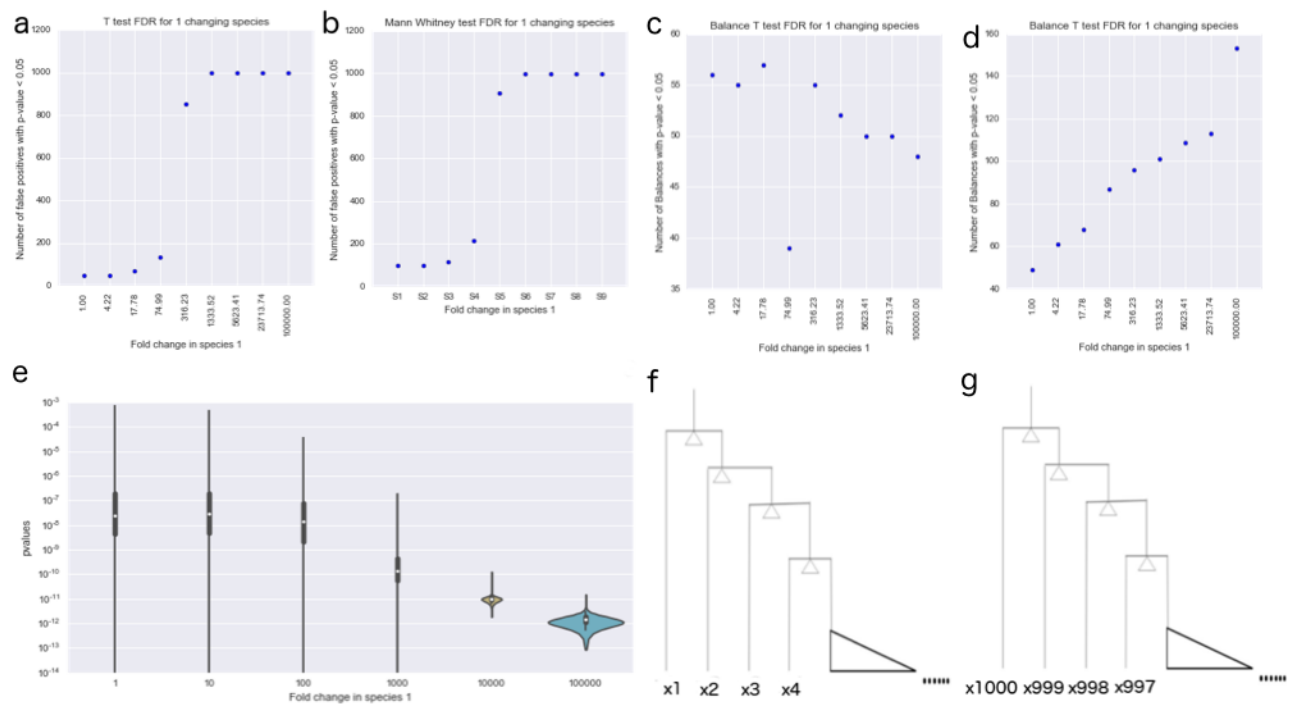

Supplement: FIG S1 [file sys001172078sf2.pdf]

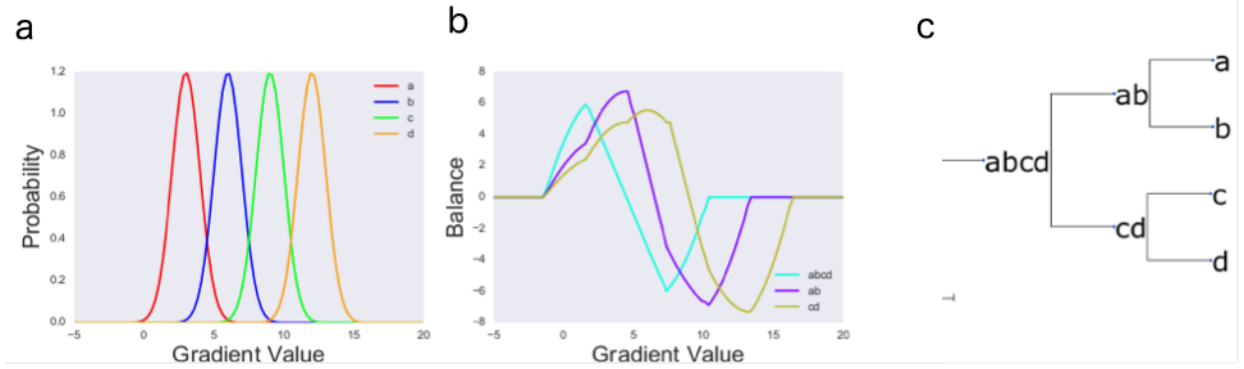

Supplement: FIG S2 [file sys001172078sf3.pdf]

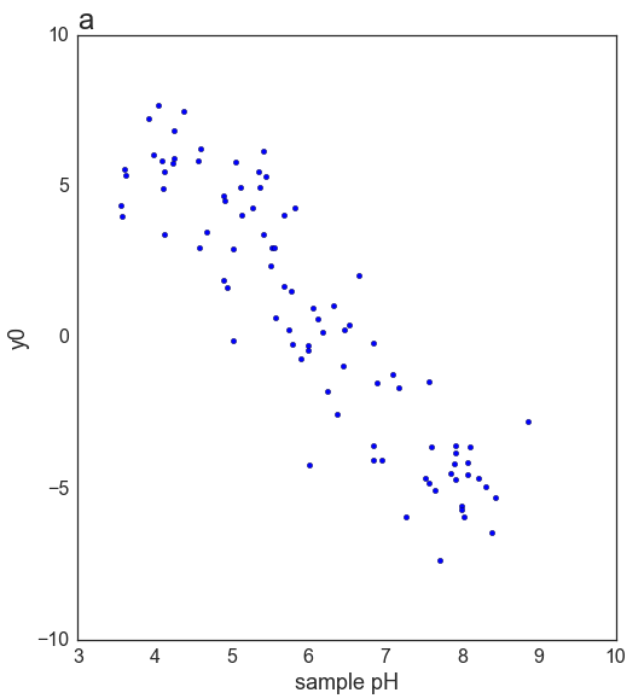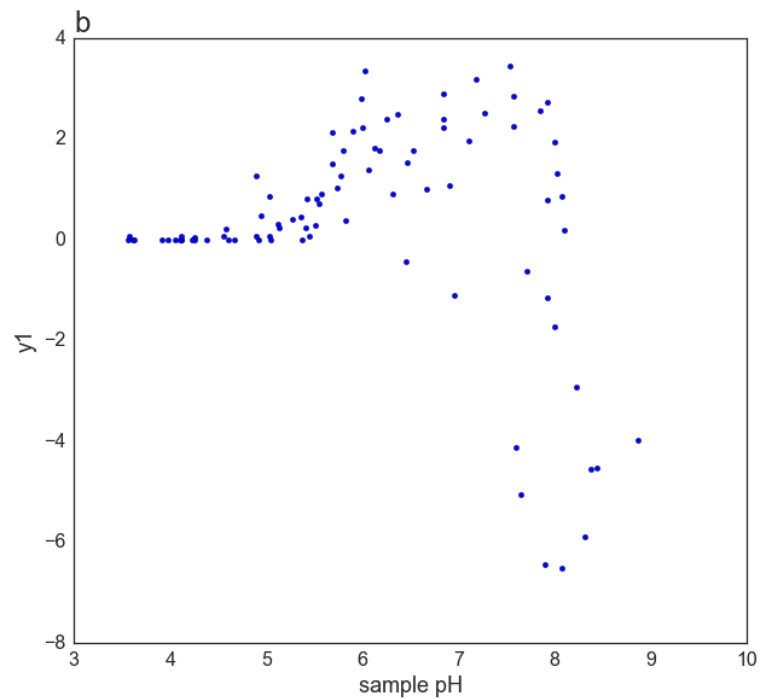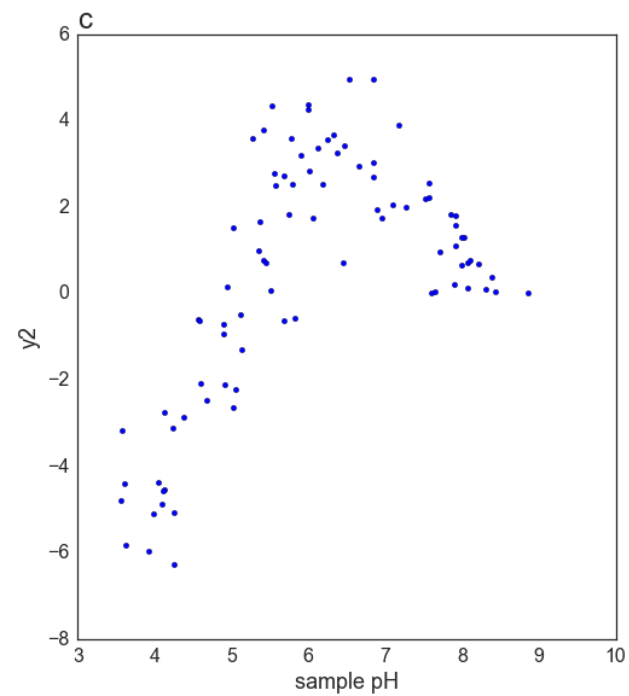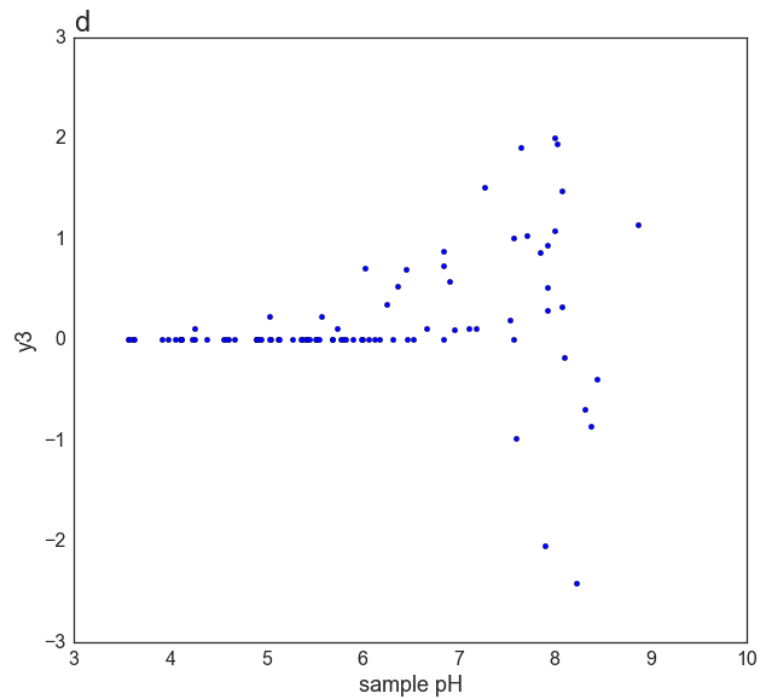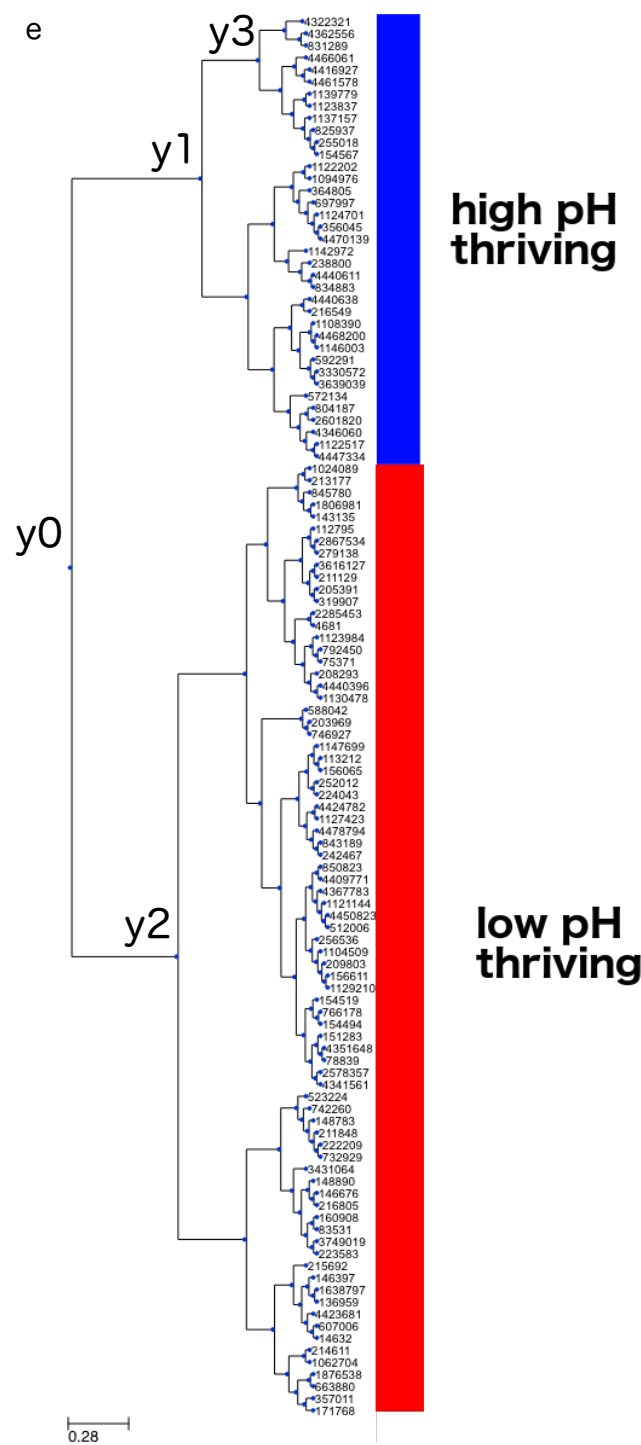

Supplement: FIG S3 [file sys001172078sf4.pdf]
