# Supplementary material for: Balance Trees Reveal Microbial Niche Differentiation
Source: mSystems. 2017 Jan 17;2(1):e00162-16. doi: 10.1128/mSystems.00162-16 (PMC5264246; doi:10.1128/mSystems.00162-16)
Supplement: TEXT S1 [file sys001172078s1.docx]

**Supplemental Material**

Equation S1

$$\log\left( \frac{\prod_{x_{j}\in i_{L}} \left( x_{j} \right)^{1/\left| i_{L} \right|}}{\prod_{x_{k}\in i_{R}} \left( x_{k} \right)^{1/\left| i_{R} \right|}} \right)=\log\left( \frac{\left( \prod_{p_{j}\in i_{L}} np_{j} \right)^{1/\left| i_{L} \right|}}{\left( \prod_{p_{k}\in i_{R}} np_{k} \right)^{1/\left| i_{R} \right|}} \right)=\log\left( \frac{\left( \prod_{p_{j}\in i_{L}} p_{j} \right)^{1/\left| i_{L} \right|}}{\left( \prod_{p_{k}\in i_{R}} p_{k} \right)^{1/\left| i_{R} \right|}} \right)=\log\left( \frac{g\left( i_{L} \right)}{g\left( i_{R} \right)} \right)$$

$n$ is the true sequencing count,$x_{j}$ is the true abundances of species j and$p_{j}$is the proportion of species j. $i_{L}$is the set of all species proportions contained in the left sub-tree at internal node $i$, $i_{R}$ is the set of all species proportions contained in the right sub-tree at the internal node $i$, and, $g\left( x \right)$ is the geometric mean of all of the proportions contained in $x$, $\left| i_{R} \right|$ is the number of species contained $i_{R}$ and $\left| i_{L} \right|$ is the number of species contained $i_{L}$. As shown above, the sequencing depth constant gets effectively canceled out. Thus, log ratios are a natural normalization for sequencing depth, especially if there are no zero abundances present and the samples have sufficient coverage.

**Supplemental Figure 1**

The simulation consisted of a uniform population of 1000 individuals. A blooming was simulated across 9 time points, where a single organism eventually grew 100,000x fold. At each time point, 30 compositions were simulated using multinomial sampling with replacement. At each time point, a statistical test was performed comparing the sample at that time point to the original time point. Since we know beforehand that only 1 species is changing, any other tests that don’t involve the first set of proportions that is determined to be significant with p-value<0.05 is a false positive Figure S1a-b). In fact, if the bloom of a given species is high enough, all of the other individuals can be detected to change. In Figure S1e, if 1 species has changed by 100,000x, then all of the pvalues will be less than 10^-10^, giving a false positive rate close to 100%. The same procedure is performed using balances, any balance that does not contain x1 that is determined to be significant from a t-test is considered a false positive (Figure S1c). Note that this is highly dependent on the choice of the tree. In this case, we used a tree where the blooming species x1 was to the far right of the tree (Figure S1f). In this way, only the balance between x1, and x2 through x1000 should be changing. But if we were to flip the tree, and place x1 to the far right of the tree, every balance in the tree will contain x1 (Figure S1g). So as x1 blooms, the number of significant balances will increase (Figure S1d).

While it may be deemed biologically irrelevant, blooms do happen frequently in microbial studies, with individual species sometimes blooming 5 orders of magnitude within a short period of time. And the 10,000x fold growth of a single species will have the exact same effect as the 1,000x fold growth change of 10 species. This suggests that there could be many subtle scenarios where we could be misinterpreting biologically relevant signals by testing individual proportions of microbes.

**Supplemental Figure 2**

A simulation of 4 species, where each species is normally distributed along some environmental gradient. Each species has a normal distribution with a variance of 3 and a mean of 3, 6, 9 and 12 respectively as shown in Figure S2a. The resulting balances can be calculated as follows

$abcd=log(\frac{\sqrt{ab}}{\sqrt{cd}})$ $ab=log(\frac{\sqrt{a}}{\sqrt{b}})$ $cd=log(\frac{\sqrt{c}}{\sqrt{d}})$

Note, it is not possible to take a logarithm of zero. A commonly used approach around this problem is to add a pseudocount. Here we add a pseudocount of 1 after multiplying all of the species probabilities by 10000. These species abundances are transformed into balances as shown in Figure S2b. Because of the zero phenomenon, the balances yield something reminiscent to a triangular wave when applied to a pair of unimodal distributions. Take balance ab for example. At the far left around -5, neither a or b are present, so both of their abundances are zero. But since we are adding pseudocounts, the resulting balance is given by $log\left( \frac{1}{1} \right)=0$ . When the gradient value increases to 0, the abundance of *a* approaches the peak of the distribution, while the abundance of *b* is still zero, causing the *ab* balance to increase. By the time the gradient value is around 4, the abundances of *b* starts to appear*,* causing the *ab* to peak. When the gradient value is around 8, the abundance of a starts disappearing while the abundance of b starts approaching the maximum peak in Figure S2a. At a gradient value of 10, the abundance of b also begins to dwindle, and the *ab* balance spirals towards zero. This same triangular wave pattern appears in all 3 of these balances, and portions of this also appears in the 88 soils study as shown in Figure S3.

It is also important to note that a balance of zero also indicates that the abundances between the ratios are equal. So if a balance is zero, and a pseudocount scheme was used, either the proportions between the numerator and denominator are truly equal, or both the numerator and the denominator are zero.

**Supplemental Figure 3**

If there is truly a unimodal species distribution along pH, we’d expect to see the same sort of triangular wave pattern as shown in Figure 2S. If this is the case, then the top balance y0 is likely to be resulting from the midsection of the triangular wave between the minimum and the maximum (Figure S3a). The peaks of the triangular wave are a bit more apparent in the Figure S3b-d. In Figure S3b, the lower subtree in y1 is probably reaching a maximum in the true abundance around a pH of 7. In Figure S3c, the upper subtree in y2 is also likely approaching a maximum in the true abundance around a pH of 6. The same sort pattern could be happening in Figure S3d with the lower subtree in y3. These glimpses of triangular waves in these graphs suggest that there could be unimodel distributions of OTUs across pH gradient.

As daunting as the zeros problem is, the zeros present in data sets such as the 88 soils follow predictable patterns. Even with a simple pseudo count strategy, we can still extract sensible information about balances of microbes across different pH values.
